# Scalable analysis of Big pathology image data cohorts using efficient methods and high-performance computing strategies

**DOI:** 10.1186/s12859-015-0831-6

**Published:** 2015-12-01

**Authors:** Tahsin Kurc, Xin Qi, Daihou Wang, Fusheng Wang, George Teodoro, Lee Cooper, Michael Nalisnik, Lin Yang, Joel Saltz, David J. Foran

**Affiliations:** 1 Department of Biomedical Informatics, Stony Brook University, Stony Brook, USA; 2 Department of Pathology & Laboratory Medicine, Rutgers -- Robert Wood Johnson Medical School, New Brunswick, USA; 3 Department of Electrical and Computer Engineering, Rutgers University, New Brunswick, USA; 4 Department of Computer Science, Stony Brook University, Stony Brook, USA; 5 Department of Computer Science, University of Brasilia, Brasília, Brazil; 6 Department of Biomedical Informatics, Emory University, Atlanta, USA; 7 Department of Biomedical Engineering, University of Florida, Gainesville, USA; 8 Rutgers Cancer Institute of New Jersey, New Brunswick, USA

**Keywords:** High performance computing, GPUs, Databases

## Abstract

**Background:**

We describe a suite of tools and methods that form a core set of capabilities for researchers and clinical investigators to evaluate multiple analytical pipelines and quantify sensitivity and variability of the results while conducting large-scale studies in investigative pathology and oncology. The overarching objective of the current investigation is to address the challenges of large data sizes and high computational demands.

**Results:**

The proposed tools and methods take advantage of state-of-the-art parallel machines and efficient content-based image searching strategies. The content based image retrieval (CBIR) algorithms can quickly detect and retrieve image patches similar to a query patch using a hierarchical analysis approach. The analysis component based on high performance computing can carry out consensus clustering on 500,000 data points using a large shared memory system.

**Conclusions:**

Our work demonstrates efficient CBIR algorithms and high performance computing can be leveraged for efficient analysis of large microscopy images to meet the challenges of clinically salient applications in pathology. These technologies enable researchers and clinical investigators to make more effective use of the rich informational content contained within digitized microscopy specimens.

## Background

Examination of the micro-anatomic characteristics of normal and diseased tissue is important in the study of many types of disease. The evaluation process can reveal new insights as to the underlying mechanisms of disease onset and progression and can augment genomic and clinical information for more accurate diagnosis and prognosis [1–3]. It is highly desirable in research and clinical studies to use large datasets of high-resolution tissue images in order to obtain robust and statistically significant results. Today a whole slide tissue image (WSI) can be obtained in a few minutes using a state-of-the-art scanner. These instruments provide complex auto-focusing mechanisms and slide trays, making it possible to automate the digitization of hundreds of slides with minimal human intervention. We expect that these advances will facilitate the establishment of WSI repositories containing thousands of images for the purposes of investigative research and healthcare delivery. An example of a large repository of WSIs is The Cancer Genome Atlas (TCGA) repository, which contains more than 30,000 tissue images that have been obtained from over 25 different cancer types.

As it is impractical to manually analyze thousands of WSIs, researchers have turned their attention towards computer-aided methods and analytical pipelines [4–12]. The systematic analysis of WSIs is both computationally expensive and data intensive. A WSI may contain billions of pixels. In fact, imaging a tissue specimen at 40x magnification can generate a color image of 100,000x100,000 pixels in resolution and close to 30GB in size (uncompressed). A segmentation and feature computation pipeline can take a couple of hours to process an image on a single CPU-core. It will generate on average 400,000 segmented objects (nuclei, cells) while computing large numbers of shape and texture features per object. The analysis of the TCGA datasets (over 30,000 images) would require 2–3 years on a workstation and generate 12 billion segmented nuclei and 480 billion features in a single analysis run. If a segmented nucleus were represented by a polygon of 5 points on average and the features were stored as 4-byte floating point numbers, the memory and storage requirements for a single analysis of 30,000 images would be about 2.4 Terabytes. Moreover, because many analysis pipelines are sensitive to input parameters, a dataset may need to be analyzed multiple times while systematically varying the operational settings to achieve optimized results.

These computational and data challenges and those that are likely to emerge as imaging technologies gain further use and adoption, require efficient and scalable techniques and tools to conduct large-scale studies. Our work contributes a suite of methods and software that implement three core functions to quickly explore large image datasets, generate analysis results, and mine the results reliably and efficiently. These core functions are:

### Function 1: Content-based search and retrieval of images and image regions of interest from an image dataset

This function enables investigators to find images of interest based not only on image metadata (e.g., type of tissue, disease, imaging instrument), but also on image content and image-based signatures. We have developed an efficient content-based image search and retrieval methodology that can automatically detect and return those images (or sub-regions) in a dataset that exhibit the most similar computational signatures to a representative, sample image patch.

A growing number of applications now routinely utilize digital imaging technologies to support investigative research and routine diagnostic procedures. This trend has resulted in a significant need for efficient content-based image retrieval (CBIR) methods. CBIR has been one of the most active research areas in a wide spectrum of imaging informatics fields [13–25]. Several domains stand to benefit from the use of CBIR including education, investigative basic and clinical research, and the practice of medicine. CBIR has been successfully utilized in applications spanning radiology [16, 23, 26, 27], pathology [21, 28–30], dermatology [31, 32] and cytology [33–35]. Several successful CBIR systems have been developed for medical applications since the 1980’s. Some of these systems utilize simple features such as color histograms [36], shape [16, 34], texture [18, 37], or fuzzy features [19] to characterize the content of images while allowing higher level diagnostic abstractions based on systematic queries [16, 37–39]. The recent adoption and popularity of case-based reasoning and evidence-based medicine [40] has created a compelling need for more reliable image retrieval strategies to support diagnostic decisions. In fact, a number of state-of-the-art CBIR systems have been designed to support the processing of queries across imaging modalities [16, 21, 23–25, 27, 28, 41–44].

Drawing from the previous work, our research and development effort has made several significant contributions in CBIR. To summarize, our team has developed **(1)** a library of image processing methods for performing automated registration, segmentation, feature extraction, and classification of imaged specimens; **(2)** data management and query capabilities for archiving imaged tissues and organizing imaging results; **(3)** algorithms and methods for automatically retrieving imaged specimens based upon similarities in computational signatures and correlated clinical data, including metadata describing the specified tissue and physical specimen; and **(4)** components for analyses of imaged tissue samples across multi-institutional environments. These algorithms, tools and components have been integrated into a software system, called *ImageMiner,* that supports a range of tissue related analyses in clinical and investigative oncology and pathology [45–49].

### Function 2: Computing quantitative features on images by segmenting objects, such as nuclei and cells, and computing shape and texture features for the delineated structures

This function gleans quantitative information about morphology of an imaged tissue at the sub-cellular scales. We have developed a framework that utilizes CPUs and GPUs in a coordinated manner. The framework enables image analysis pipelines to exploit the hybrid architectures of modern high-performance computing systems for large-scale analyses.

The use of CPU-GPU equipped computing systems is a growing trend in the high performance computing (HPC) community [50]. Efficient utilization of these machines is a significant problem that necessitates new techniques and software tools that can optimize the scheduling of application operations to CPUs and GPUs. This challenge has motivated several programming languages and runtime systems [51–62] and specialized libraries [63]. Ravi et al. [60, 61] proposed compiler techniques coupled with runtime systems for execution of generalized reductions in CPU-GPU machines. Frameworks such as DAGuE [59] and StarPU [54] support regular linear algebra applications on CPU-GPU machines and implement scheduling strategies that prioritize computation of tasks in the critical path of execution. The use of HPC systems in Biomedical Informatics research is an increasingly important topic, which has been the focus of several recent research initiatives [46, 48, 57, 58, 64–70]. These efforts include GPU-accelerated systems and applications [71–78].

A major concentration for our team has been the development of algorithms, strategies and tools that facilitate high-throughput processing of large-scale WSI datasets. We have made several contributions in addressing and resolving several key issues: **(1)** Some image analysis operations have regular data access and processing patterns, which are suitable for parallelization on a GPU. However, data access and processing patterns in some operations, such as morphological reconstruction and distance transform operations in image segmentation, are irregular and dynamic. We have developed a novel queue based wavefront propagation approach to speed up such operations on GPUs, multi-core CPUs, and combined CPU-GPU configurations [79]; **(2)** Image analysis applications can be implemented as hierarchical data flow pipelines in which coarse-grain stages are divided into fine-grain operations. We have developed runtime to support composition and execution of hierarchical data flow pipelines on machines with multi-core CPUs and multiple GPUs [80, 81]. Our experiments showed significant performance gains over single program or coarse-grain workflow implementations; **(3)** Earlier work in mapping and scheduling of application operations onto CPUs and GPUs primarily targeted regular operations. Operations in image analysis pipelines can have high variability with respect to GPU accelerations and their performances depend on input data. Hence, high throughput processing of datasets on hybrid machines requires more dynamic scheduling of operations. We have developed a novel priority-queue based approach that takes into account the variability in GPU acceleration of data processing operations to perform better scheduling decisions [80, 81]. This seemingly simple priority-queue data structure and the associated scheduling algorithm (which we refer to as performance aware scheduling technique) have showed significant performance improvements. We have combined this scheduling approach with other optimizations such as data locality aware task assignment, data prefetching, and overlapping of data copy operations with computations in order to reduce data copy costs. **(4)** We have integrated all of these optimizations into an operational framework.

### Function 3: Storing and indexing computed quantitative features in a database and mining them to classify images and subjects

This function enables interrogation and mining of large volumes of analysis results for researchers to look for patterns in image data and correlate these signatures with profiles of clinical and genomic data types. We have developed methods that leverage HPC and Cloud database technologies to support indexing and querying large volumes of data. Clustering algorithms use heuristics for partitioning a dataset into groups and are shown to be sensitive to input data and initialization conditions. Consensus and ensemble clustering approaches aim to address this problem by combining results from multiple clustering runs and/or multiple algorithms [82–89]. This requires significant processing power and large memory space when applied to large numbers of segmented objects. For example, when classification and correlation analyses are carried out on an ensemble of segmented nuclei, the number of delineated objects of interest may rise well into the millions. We introduce a parallel implementation of consensus clustering on a shared-memory cluster system to scale the process to large numbers of data elements for image analysis applications.

## Methods

**Fig. 1:**
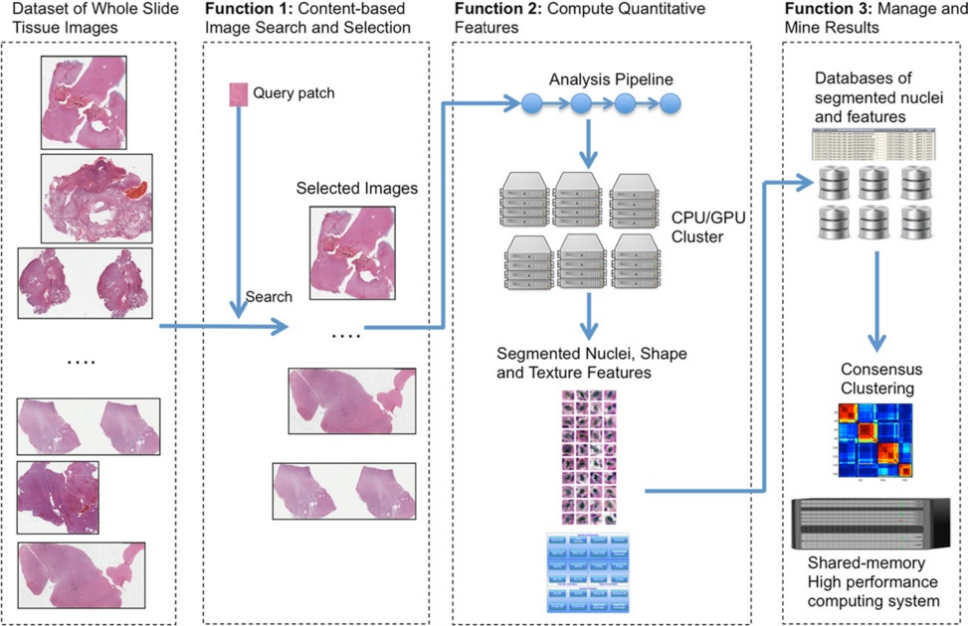
Functions supported by methods and tools described in this paper. Starting from a dataset of whole slide images, a researcher can employ methods in Function 1 to select a subset of images based on image content. If an image has a patch that is similar to the query patch, the image is selected for processing. The selected set of images is then processed through analysis pipelines in Function 2. In the figure, the analysis pipeline segments nuclei in each image and computes a set of shape and texture features. The segmented nuclei and their features are loaded to a database for future analyses in Function 3. In addition, the researcher runs a clustering algorithm to cluster nuclei and patients into groups to look at correlations with groupings based on clinical and genomic data. Clustering, more specifically consensus clustering, requires significant memory and computation power. Using the methods described in this paper, the research can employ a shared-memory system to perform consensus clustering

Figure 1 illustrates the three functions we have introduced in Section 1 using a high-level image analysis example. In this scenario, an investigator is interested in studying relationships between the properties of nuclei and clinical and molecular data for tissues that exhibit features similar to those of Gleason grade 5 prostate cancer tissue images (see Section 2.1 for a description of those features). **Function 1:** The investigator searches for images in a dataset of WSIs based not only on image metadata, e.g., prostate cancer tissue, but also based on whether an image contains patches that are similar to a given image query patch. The image query patch is a small image tile containing representative tissue with the target Gleason grade. In Section 2.1, we describe the methodologies to perform quick, reliable CBIR. We presented a high-throughput parallelization approach for CBIR algorithms in earlier work [49, 90]. **Function 2:** The output from Function 1 is a set of images and imaged regions that exhibit architecture and staining characteristics which are consistent with the input query provided by the investigator. In order to extract refined morphological information from the images, the investigator composes an analytical pipeline consisting of segmentation and feature computation operations. The segmentation operations detect nuclei in each image and extract their boundaries. The feature computation operations compute shape and texture features, such as area, elongation, and intensity distribution, for each segmented nucleus. The investigator runs this pipeline on a distributed memory cluster to quickly process the image set. We describe in Section 2.2 a framework to take advantage of high performance computing systems, in which computation nodes have multi-core CPUs and one or more GPUs, for high throughput processing of a large number of WSIs. **Function 3:** The analysis of images can generate a large number of features –the number of segmented nuclei in a single analysis run can be hundreds of millions for a dataset of a few thousand images. The investigator stores the segmentation results in a high performance database for further analysis. The next step after the segmentation and feature computation process is to execute a classification algorithm to cluster the segmentation results into groups and look for correlations between this grouping and the grouping of data based on clinical and molecular information (e.g., gene mutations or patient survival rates). A consensus clustering approach may be preferable to obtain robust clustering results [91]. In Section 2.3, we present a consensus clustering implementation for shared-memory parallel machines.

### Function 1. Efficient methodology to search for images and image regions: hierarchical CBSIR

This function facilitates the retrieval of regions of interests within an image dataset. These regions have similar color, morphology or structural patterns to those of the query image patch. In this way, investigators and physicians can select a set of images based on their contents in addition to any corresponding metadata such as the type of tissue and image acquisition device utilized. In this function, given a query patch, each image within the data set is scanned in x and y directions systematically detecting each image patch having the patterns of the query patch [90]. For each candidate image patch, a CBIR algorithm is applied to check if the image patch is similar to the query patch. Existing CBIR libraries are mostly focused on natural or computer vision image retrieval. Our CBIR algorithm is primarily designed to support the interrogation and assessment of imaged histopathology specimens.

This approach is based on a novel method called hierarchical annular feature (HAF) and a three-stage search scheme. It provides several benefits: (1) scale and rotation invariance; (2) capacity to capture spatial configuration of image local features; and (3) suitability for hierarchical searching and parallel sub-image retrieval.

#### Execution of CBIR process

The CBIR process is executed in three stages: hierarchical searching, refined searching and mean-shift clustering. The hierarchical searching stage is an iterative process that discards those candidates exhibiting signatures inconsistent with the query. This is done in step-wise fashion in each iteration. The stage begins by calculating the image features of the inner (first) central bins for candidate patches and compares them with those of the query patch. Based on the level of dissimilarity between the query patch and the patch being tested, it removes a certain percentage of candidates after the first iteration. For the second iteration, it calculates the image features from the second central bin only, and further eliminates a certain percentage of candidates by computing the dissimilarity with the features of the query patch from the two inner bins. At the end of this stage, the final candidates are those that have passed all prior iterations. These are the candidates that are most similar to the query patch. The final results are further refined by computing image features from 8-equally-divided segments of each annular bin. To rank the candidates in each step, dissimilarity between the query’s and the candidate patches’ features is defined as their Euclidean distances. The hierarchical searching procedure can greatly reduce the time complexity, because it rejects a large portion of candidates in each iteration. The number of candidates moving to the next step is significantly reduced by rejecting the obvious negative candidates. In the refined searching stage, each annular bin is equally divided into 8 segments, and image features in each segment is computed and combined to generate one single feature vector. Due to the very limited number of candidates passing the hierarchical searching stage, this refined process is not particularly time consuming. In the last stage, a mean-shift clustering is applied to generate the final searching results.

#### Description of the Computation of Hierarchical Annular Features (HAF)

**Fig. 2:**
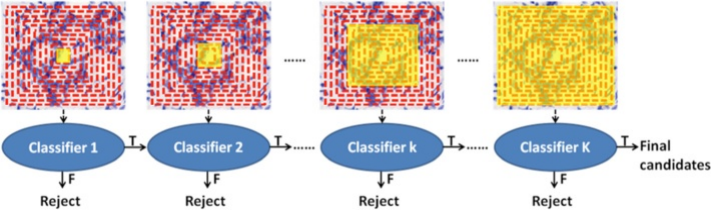
Content-based image search using hierarchical annular features (HAF). In the first stage of the search operation, an iterative process is carried out in which a percent of candidate images or image patches are discarded in each iteration. At the end of this stage, successful candidates are refined in the second stage of processing

A given image is segmented into several closed bins with equal intervals, as shown in Fig. 2. Next, image feature of each bin is computed and then all the image features are concatenated to form a single vector, which we call hierarchical annular feature (HAF). With HAF, the discriminative power of each image patch descriptor is significantly improved compared with traditional image features extracted from the whole image. For medical images, it is very likely that image patches containing different structures have quite similar intensity distribution as a whole, but yet exhibit different HAF signatures.

For the study reported in this paper, we focused on a representative application focused on prostate cancer. Prostate cancer is the second leading cause of male deaths in the U.S. with over 230,000 men diagnosed annually. Gleason scoring is the standard method for stratifying prostate cancer from onset of malignancy through advanced disease. Gleason grade 3 typically consists of infiltrative well-formed glands, varying in size and shapes. Grade 4 consists of poorly formed, fused or cribriform glands. Grade 5 consists of solids sheets or single cells with no glandular formation. In recognition of the complexity of the different Gleason grades, we utilized two different resolution image features to capture the characteristics of the underlying pathology of an ensemble of digitized prostate specimens. At low-magnification (10X), texture features are extracted to identify those regions with different textural variance. At high-magnification (20X), structural features are characterized. This strategy takes advantage of sampling patches from the whole-slide image while generating feature quantification at two different resolutions. This approach effectively minimizes the computation time while maintaining a high level of discriminatory performance. Section 3.1 provides the details of the steps taken to achieve automated analysis of imaged prostate histology specimens for the purposes of performing computer-assisted Gleason grading.

#### Texture features

The Gabor filter is a widely used because of its capacity to capture image texture characteristics at multiple directions and resolutions. In our work, we use 5 scales, and 8 directions to build the Gabor filter set. The mean and variance extracted from the filtered image are used to build an 80-dimension feature vector.

#### Structural features

**Fig. 3:**
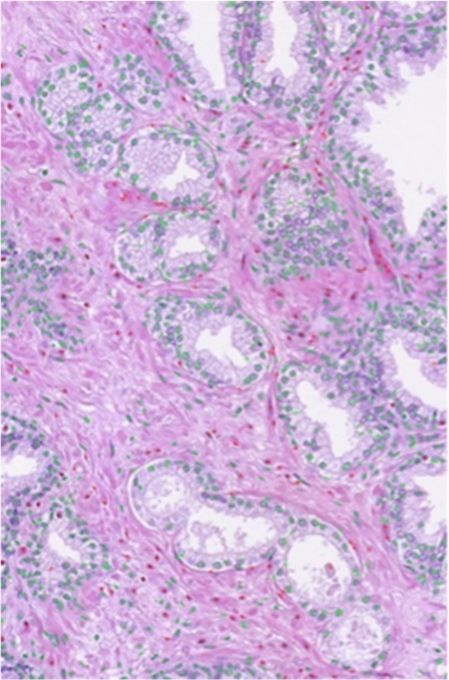
Detected glandular nuclei (*rendered with green cross*) and stromal region nuclei (*labeled with red-crosses*) during the content-based image search and retrieval process

In addition to the spatial structural differences, the density of nuclei on glands within the tissue samples increases during the course of disease progression which is reflected in the assignment of higher Gleason grades. In the case of Grade 5 images, however, only the nuclei and cytoplasm are evident with no clear glandular formation. The algorithms that we developed perform color segmentation to classify ach pixel as nuclear, lumen, cytoplasm or stroma. Figure 3 illustrates the glandular nuclei (green cross labeled) and stromal region nuclei (red cross labeled).

### Function 2. High throughput computation of quantitative measures on hybrid CPU-GPU systems

In most pathology imaging applications it is necessary to process image sets using a pipeline which performs image segmentation and a series of computational stages to generate the quantitative image features used in the analysis. In this paper, we introduce a nuclear segmentation pipeline. In the segmentation stage, nuclei in the image are automatically detected and their boundaries are extracted. The feature computation stage computes shape and texture features including area, elongation and intensity distribution or each segmented nucleus. These features may then be processed in a classification stage to cluster nuclei, images and patients into groups. We provide a detailed description of the high-performance computing approaches used for managing and clustering segmented nuclei in Section 2.3. In this section, we present approaches to enable high throughput processing of a large set of WSIs in a pipeline of image segmentation and feature computation stages. The goal is to reduce the execution times of the pipeline from days to hours and from weeks to days, when hundreds or thousands of images are to be analyzed.

Modern high performance computing systems with nodes of multi-core CPUs and co-processors (i.e., multiple GPUs and/or Intel Xeon Phi’s) offer substantial computation capacity and distributed memory space. We have devised a framework, which integrates a suite of techniques, optimizations, and a runtime system, to take advantage of such systems to speed up processing of large numbers of WSIs [81]. Our framework implements several optimizations at multiple levels of an analytical pipeline. This includes optimizations in order to execute irregular pipeline operations on GPUs efficiently and scheduling of multi-level pipelines of operations to CPUs and GPUs in coordination to enable rapid processing of a large set of WSIs. The runtime system is designed to support high-throughput processing through a combined bag-of-tasks and dataflow computation pattern. We have chosen this design because of the characteristics of WSI data and WSI analysis pipelines as we describe below.

A possible strategy for speeding up segmentation of nuclei on a multi-core CPU is to parallelize each operation to run on multiple CPU cores. The processing of an image (or image tile) is partitioned across multiple cores. When a nucleus is detected, the segmentation of the nucleus is also carried out on multiple cores. This parallelization strategy is generally more applicable for a data set that has a relatively small number of large objects, because synchronization and data movement overheads can be amortized. A nucleus, however, occupies a relatively small region (e.g., 8x8 pixels) compared with the entire image (which can be 100Kx100K pixels). We performed micro-benchmarks on CPUs and GPUs to investigate performance with respect to atomic operations, random data accesses, etc. For instance, in a benchmark for atomic operations using all the cores available on the GPU and on the CPU, the GPU was able to execute close to 20 times more operations than the CPU. When we evaluated the performance of random memory data reads, the GPU was again faster -- as it performed up to 895 MB/s vs. 305 MB/s for the multi-core CPU in reading operations. These benchmarks showed that the GPU is more suitable than the CPU for finer-grain parallelization in our image analysis pipelines and that a coarser grain parallelization would be better for the CPU. While a nucleus is small, an image may contain hundreds of thousands to millions of nuclei. Moreover, we target datasets with hundreds to thousands of images. For execution on CPUs, an image level parallelization with a bag-of-tasks execution model will be more suitable for these datasets than a parallelization strategy that partitions the processing of a nucleus across multiple CPU-cores. Thus, in our framework, each image in a dataset is partitioned into rectangular tiles. Each CPU core is assigned an image tile and the task of segmenting all the nuclei in that image tile – we have not developed multi-core CPU implementations of operations in this work for this reason. Multiple image tiles are processed concurrently on multiple cores, multiple CPUs, and multiple nodes, as well as on multiple GPUs when a node has GPUs. This approach is effective because even medium size datasets with a few hundred images can have tens of thousands of image tiles and tens of millions of nuclei.

For execution on GPUs, we have leveraged existing implementations from the OpenCV library [63] and other research groups [92, 93] whenever possible. When no efficient implementations were available, we developed them in-house [79–81]. In our case, one of the challenges to the efficient use of a GPU is the fact that many operations in the segmentation stage are irregular and, hence, are more challenging to execute on a GPU. Data access patterns in these operations are irregular (random), because only active elements are accessed, and those elements are determined dynamically during execution. For instance, several operations, such as Reconstruct to Nucleus, Fill Holes, and Pre-Watershed, are computed using a flood fill scheme proposed by Vincent [92]. This scheme in essence implements an irregular wavefront propagation in which active elements are the pixels in wavefronts. We have designed and implemented an efficient hierarchical parallel queue to support execution of such irregular operations on a GPU. While the maintenance of this queue is much more complex than having a sequential CPU-based queue, comparisons of our implementations to the state-of-the art implementations show that our approach is very efficient [79]. Other operations in the segmentation stage, such as Area Threshold and Black and White Label, are parallelized using a Map-Reduce pattern. They rely on the use of atomic instructions, which may become a bottleneck on GPUs and multi-core CPUs. Operations in the feature computation stage are mostly regular and have a high computing intensity. As such, they are more suited for efficient execution on GPUs and expected to attain higher GPU speedups. We have used CUDA^1^ for all of the GPU implementations. The list of the operations in our current implementation is presented in Tables 1 and 2. The sources of the CPU and GPU implementations are presented in their respective columns of the table.

**Table 1:** The list of operations in the segmentation and feature computation stages and the sources of the CPU and GPU versions

Segmentation Computations		
Operation	CPU Implementation	GPU Implementation
Red Blood Cell Detection (RBC Detection)	Vincent [92] and OpenCV	Implemented
Morphological Open (Morph. Open)	OpenCV	OpenCV
Reconstruct to Nucleus (ReconToNuclei)	Vincent [92]	Implemented
Area Threshold	Implemented	Implemented
Fill Holes	Vincent [92]	Implemented
Pre-Watershed	Vincent [92], and OpenCV for distance transformation	Implemented
Watershed	OpenCV	Körbes [93]
Black and White Label (BWLabel)	Implemented	Implemented

We used the implementation of the morphological reconstruction operation by Vincent for the implementation of several segmentation operations. *Implemented* indicates our implementation of the respective operations

**Table 2:** The list of operations in the segmentation and feature computation stages and the sources of the CPU and GPU versions

Feature Computations			
Class	Operations	Computed Features	CPU and GPU Implementation
Pixel Statistics	Histogram Calculation	Mean, Median, Min, Max, 25 %, 50 %, and 75 % quartile	Implemented
Gradient Statistics	Gradient and Histogram Calculation	Mean, Median, Min, Max, 25 %, 50 %, and 75 % quartile	Implemented
Haralick	Normalization pixel values and Co-occurrence matrix	Inertia, Energy, Entropy, Homogeneity, Max prob, Cluster shade, Prominence	Implemented
Edge	Canny and Sobel	Canny area, Sobel area	OpenCV (Canny), Implemented (Sobel)
Morphometry	Pixel counting, Dist. among points, Area and Perimeter, Fitting ellipse, Bounding box, Convex hull, Connected components, Area, Perimeter, Equivalent diameter, Compactness, Major/Minor axis length, Orientation, Eccentricity, Aspect ratio, Convex area, Euler number	Implemented	

We used the implementation of the morphological reconstruction operation by Vincent for the implementation of several segmentation operations. *Implemented* indicates our implementation of the respective operations

Even though not all operations (e.g., irregular operations) in an analysis pipeline map perfectly to GPUs, most modern high performance machines come with one or more GPUs as co-processing units. Our goal is to leverage this additional processing capacity as efficiently as possible. We should note that the runtime system of our framework does not use only GPUs on a machine. As we shall describe below, it coordinates the scheduling of operations to CPU cores and GPUs to harvest the aggregate computation capacity of the machine. Each image tile is assigned to an idle CPU core or GPU; multiple CPU cores and GPUs process different input tiles concurrently.

**Fig. 4:**
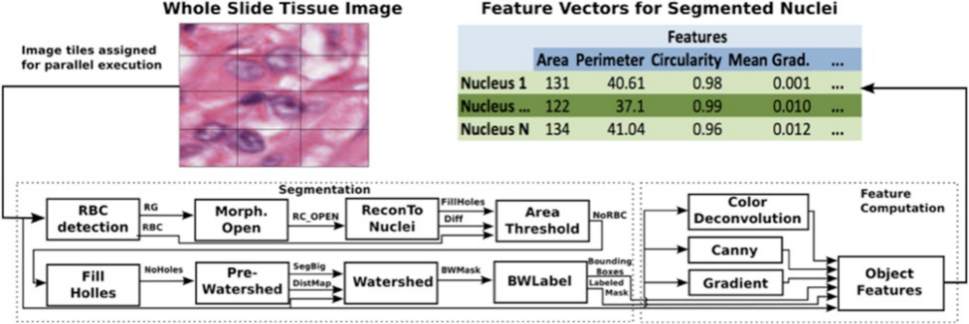
Pipeline for segmenting nuclei in a whole slide tissue image, and computing a feature vector of characteristics per nucleus. The input to the pipeline is an image or image tile. The output is a set of features for each segmented nucleus. The segmentation stage consists of a pipeline of operations that detect nuclei and extract the boundary of each nucleus – please see Tables 1 and 2 for the full names of the operations. Each segmented nucleus is processed in the feature computation stage to compute a set of shape and texture features. The features include circularity, area, mean gradient of intensity (please see Tables 1 and 2 for the types and names of the features) as shown in the figure

**Fig. 5:**
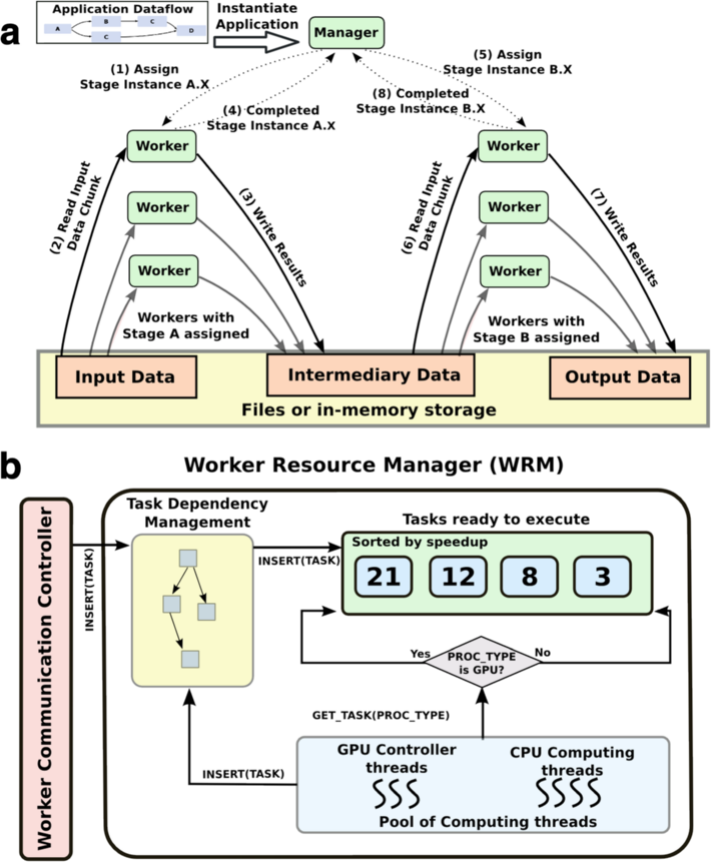
Strategy for high throughput processing of images. (**a**) Execution on multiple nodes (*left*) is accomplished using a Manager-Worker model, in which stage tasks are assigned to Workers in a demand-driven fashion. A stage task is represented as a tuple of (stage name, data). The stage name may be “segmentation” in which case data will be an image tile, or it may be “feature computation” in which case data will be a mask representing segmented nuclei in an image tile and the image tile itself. The runtime system schedules stage tasks to available nodes while enforcing dependencies in the analysis pipeline and handles movement of data between stages. A node may be assigned multiple stage tasks. (**b**) A stage task scheduled to a Worker (*right*) is represented as a dataflow of operations for the segmentation stage and a set of operations for the feature computation stage. These operations are scheduled to CPU cores and GPUs by the Worker Resource Manager (WRM). The WRM uses the priority queue structure (shown as “sorted by speedup rectangle” in the figure) to dynamically schedule a waiting operation to an available computing devices

The runtime system employs a Manager-Worker model (Fig. 5(a)) to implement the combined bag-of-tasks and dataflow pattern of execution. There is one Manager, and each node of a parallel machine is designated as a Worker. The processing of a single image tile is formulated as a two-level coarse-grain dataflow pattern. Segmentation and feature computation stages are the first level, and the operations invoked within each stage constitute the second level (Fig. 4). The segmentation stage itself is organized into a dataflow graph. The feature computation stage is implemented as a bag-of-tasks, i.e., multiple feature computation operations can be executed concurrently on segmented objects. This hierarchical organization of an analysis pipeline into computation stages and finer-grain operations within each stage is critical to the efficient use of nodes with CPUs and GPUs, because it allows for more flexible assignment of finer-grain operations to processing units (CPU cores or GPUs) and, hence, better utilization of available processing capacity.

The Manager creates instances of the segmentation and feature computation stages, each of which is represented by a *stage task*: (image tile, processing stage), and records the dependencies between the instances to enforce correct execution. The stage tasks are scheduled to the Workers using a demand-driven approach. When a Worker completes a stage task, it requests more tasks from the Manager, which chooses one or more tasks from the set of available tasks and assigns them to the Worker. A Worker may ask for multiple tasks from the Manager in order to keep all the computing devices on a node busy. Local Worker Resource Manager (WRM) (Fig. 5(b)) controls the CPU cores and GPUs used by a Worker. When the Worker receives a stage task, the WRM instantiates the finer-grain operations comprising the stage task. It dynamically creates *operation tasks*, represented by a tuple (input data, operation), and schedules them for execution as it resolves the dependencies between the operations – operations in the segmentation stage form a pipeline and operations in the feature computation stage depend on the output of the last operation in the segmentation stage.

The set of stage tasks assigned to a Worker may create many operation tasks. The primary problem is to map operation tasks to available CPU cores and GPUs efficiently to fully utilize the computing capacity of a node. Our runtime system addresses this mapping and scheduling problem in two ways. First, it makes use of the concept of function variants [51, 94]. A function variant represents multiple implementations of a function with the same signature – the same function name and the same input and output parameters. In our case, the function variant corresponds to the CPU and GPU implementations of each operation. When an operation has only one variant, the runtime system can restrict the assignment of the operation to the appropriate type of computing device. Second, the runtime system executes a performance aware scheduling strategy to more effectively schedule operations to CPUs and GPUs for the best aggregate analysis pipeline performance. Several recent efforts [51–54] have worked on the problem of partitioning and mapping tasks between CPUs and GPUs for applications in which all operations have similar GPU speedups. In our case, operations in the segmentation and feature computation phases have diverse computation and data access patterns. As a result, the amount of acceleration on a GPU varies across the operations. We have developed a task scheduling strategy, called Performance Aware Task Scheduling (PATS), which assigns tasks to CPU cores or GPUs based on an estimate of each task’s GPU speedup and on the computational loads of the CPUs and GPUs [80, 81]. The scheduler employs a demand-driven approach in which devices (CPU-cores and GPUs) request tasks as they become idle. It uses a priority queue of operation tasks, i.e., (data element, operation) tuples, sorted based on the expected amount of GPU acceleration of each tuple. New task tuples are inserted into the queue such that the queue remains sorted (see Fig. 5(b)). When a CPU core or a GPU becomes idle, one of the tuples from the queue is assigned to the idle device. If the idle device is a CPU core, the tuple with the minimum estimated speedup value is assigned to the CPU core. If the idle device is a GPU, the tuple with the maximum estimated speedup is assigned to the GPU. The priority queue structure allows for dynamic assignment of tasks to appropriate computing devices with a small maintenance overhead. Moreover, PATS relies on the order of tasks in the queue rather than the accuracy of the speedup estimates of individual tasks. As long as inaccuracy in speedup estimates is not large enough to affect the task order in the queue, PATS will correctly choose and map tasks to computing devices.

The cost of data transfer between the CPU and the GPU reduces the benefits of using the GPU. We have extended the base scheduler to facilitate data reuse. In addition to the extension for data reuse, we have implemented pre-fetching and asynchronous data copy to further reduce data transfer overheads [95].

### Function 3. Managing and mining quantitative measures

After nuclei have been segmented and their features computed, a research study will require storage and management of the results for future analyses. It will employ machine learning and classification algorithms in order to look for relationships between tissue specimens and correlations of image features with genomic and clinical data. In this section we provide an overview of our work to address challenges in managing large volumes of segmented objects and features and in carrying out consensus clustering of large volumes of results.

#### Data management

We leverage emerging database technologies and high performance computing systems in order to scale data management capabilities to support large scale analysis studies.

We have developed a detailed data model to capture and index complex analysis results along with metadata about how the results were generated [96]. This data model represents essential information about images, markups, annotations, and provenance. Image data components capture image reference, resolution, magnification, tissue type, disease type as well as metadata about image acquisition parameters. For image markups, in addition to basic geometric shapes (such as points, rectangles, and circles), polygons and polylines as well as irregular image masks are also supported. Annotations could be human observations, machine generated features and classifications. Annotations can come with different measurement scales and have a large range of data types, including scalar values, arrays, matrixes, and histograms. Comparisons of results from multiple algorithms and/or multiple human observers require combinations of metadata and spatial queries on large volumes of segmentations and features. The data model is supported by a runtime system which is implemented on a relational database system for small-to-moderate scale deployments (e.g., image datasets containing up to a hundred images) and on a Cloud computing framework for large scale deployments (involving thousands of images and large numbers of analysis runs) [97]. Both these implementations enable a variety of query types, ranging from metadata queries such as “Find the number of segmented objects whose feature f is within the range of a and b” to complex spatial queries such as “Which brain tumor nuclei classified by observer O and brain tumor nuclei classified by algorithm P exhibit spatial overlap in a given whole slide tissue image” and “What are the min, max, and average values of distance between nuclei of type A as classified by observer O”.

#### Consensus clustering on large shared-memory systems

Clustering is a common data mining operation [98]. Clustering algorithms employ heuristics and are sensitive to input parameters. A preferred mechanism is the consensus clustering approach to reduce sensitivity to input data and clustering parameters and obtain more reproducible results [91]. In consensus clustering, multiple runs of clustering algorithms on a dataset are combined to form the final clustering results. This process is computationally expensive and requires large memory space when applied to a large number of objects and features.

Our implementation is based on the method proposed by Monti et. al. [91] and consists of the following main steps: *sampling, base clustering, construction of a consensus matrix, clustering of the consensus matrix,* and *mapping*. The sampling step extracts N data points (i.e., nuclei and cells) from the entire dataset via sampling. In the second step, a clustering algorithm, e.g., K-means [99, 100], is executed *M* times with different initial conditions. The third step constructs a consensus matrix from the M runs. The consensus matrix is an NxN matrix. The value of element (*i,j*) indicates the number or percentage of the clustering runs, in which the two data points *i* and *j* were in the same cluster. In the fourth step, the consensus matrix is clustered to produce the final clustering result. The matrix is conceptually treated as a dataset of N data points, in which each data point has N dimensions. That is, each row (or column) of the matrix is viewed as a data point, and the row (or the column) values correspond to the values of the N dimensional vector of the data point. The last step maps the data points that were not selected in the sampling step to the final set of clusters. Each data point is mapped to the center of the closest cluster.

We focused on the base clustering, consensus matrix construction and consensus matrix clustering steps, since they are the most expensive. We use a publicly available parallel k-means algorithm [101] as our base clustering algorithm. In our implementation, the base clustering step is executed M times consecutively with one instance of the k-means algorithm using all of the available cores at each run. To construct the consensus matrix, the rows of the matrix are partitioned evenly among CPU cores. To compute a row *i* of the consensus matrix, the CPU core to which row *i* is mapped reads the base clustering results, which are stored in the shared memory, computes the number of times data points (*i,j*) are in the same cluster, and updates row *i*. The consensus matrix is a sparse matrix. When all M base clustering runs have been processed, row *i* is compressed using run length encoding to reduce memory usage. Multiple rows of the consensus matrix are computed concurrently. The clustering of the consensus matrix is carried out using the parallel k-means algorithm. We have modified the k-means implementation so that it works with compressed rows.

## Results and discussion

We present an evaluation of the methods described in Section 2 using real image datasets. The results are organized into three subsections with respected to the three core functions.

### Function 1: CBIR performance: speed and accuracy

In this section we present an experimental evaluation of the CBIR speed and accuracy performance using a dataset of prostate cancer images. To avoid the pitfalls of developing the tools in isolation and then later evaluating them in a clinical setting, we work closely with oncologists and pathologists and test and optimize performance throughout the course of the development cycle.

In the first phase of the project we utilized the TMA analysis tools to investigate the effect of therapeutic starvation on prostate cancer by quantifying Beclin1 staining characteristics. Mixed sets of new TMA’s were prepared with antibody for Beclin 1 and antibodies for androgen co-factor MED1, high-molecular weight keratin 34BE12, p63, and alpha methyl-Co A racemase (P504S AMACR).

To validate the proposed CBIR algorithm, we tested it on a dataset of 530 prostate histopathology images. The dataset was collected from 28 cases of whole slide imaging (WSI) from University of Iowa School of Medicine and University of Pittsburgh Medical Center (UPMC), with pixel resolution of 4096x4096 at 20X optical magnification, and 2048x2048 at 10X optical magnification. In consideration of the average query patch size for prostate gland representation in the given magnification, we use 5 bins in the HAF feature, and 50 % overlap percentage during the hierarchical searching, thus the HAF feature would capture enough content information in the underlying pathology while keep the computation amount within reasonable range.

**Fig. 6:**
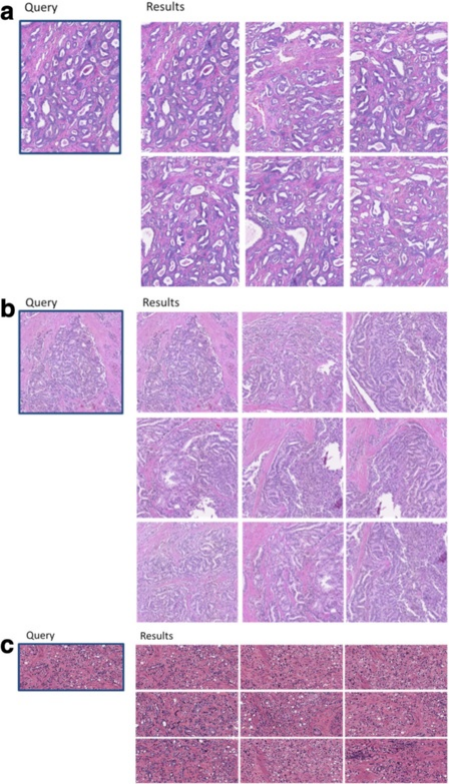
An example set of prostate Gleason grade query patches (left) and sets of matching image patches in a given set of images (right). **a** Gleason grade 3 query patch and matching image regions. **b** Gleason grade 4 query patch and matching image regions. **c** Gleason grade 5 query patch and matching image regions

To test the performance of the algorithm on prostate images of different Gleason grades, we conducted our experiments using randomly selected query patches of Gleason grade 3, 4 and 5. Figure 6 shows representative examples for prostate Gleason grade 3 (a), 4 (b) and 5 (c) query images retrieval results respectively.

To further evaluate the accuracy of the CBIR algorithm, the recall rate from the top 100 retrieved patches was calculated. We define the recall rate as

$$ recallRate=\frac{Total\  relevant\  results\  retrieved}{All\  relevant\  patches\  exists\  in\  the\  topN\  range} $$

**Fig. 7:**
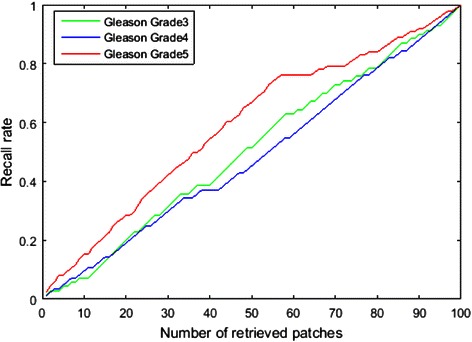
Average recall rate curves of Gleason grades 3, 4 and 5, respectively

We define a retrieved result “relevant” if it has the same Gleason grade as the query image. We use top 100 as the calculation range. The average recall rate curves of Gleason grade 3, 4 and 5 are showed in Fig. 7.

### Function 2: high performance computation of quantitative measures

The methods and tools to support function 2 were evaluated on a distributed memory parallel machine called Keeneland [50]. Each Keeneland node has a dual 6-core Intel X5660 CPUs, 24GB RAM, and 3 NVIDIA M2090 GPUs. The nodes are connected using a QDR Infiniband switch. The image datasets used in the evaluation had been obtained in brain tumor studies [2]. Each image was partitioned into tiles of 4 K × 4 K pixels. The experiments were repeated 3 times**;** the standard deviation was not higher than 2 %. The speedups were calculated based on the single CPU core versions of the operations. The CPU codes were compiled using “gcc 4.1.2” with the “-O3” optimization as well as the vectorization option to let the compiler auto-vectorize regular operations, especially in the feature computation phase. The GPU codes were compiled using CUDA 4.0. The OpenCV 2.3.1 library was used for the operations based on OpenCV. Our codes are publicly available as Git repositories,^2^^.^ 3

#### Performance of GPU-enabled operations

**Fig. 8:**
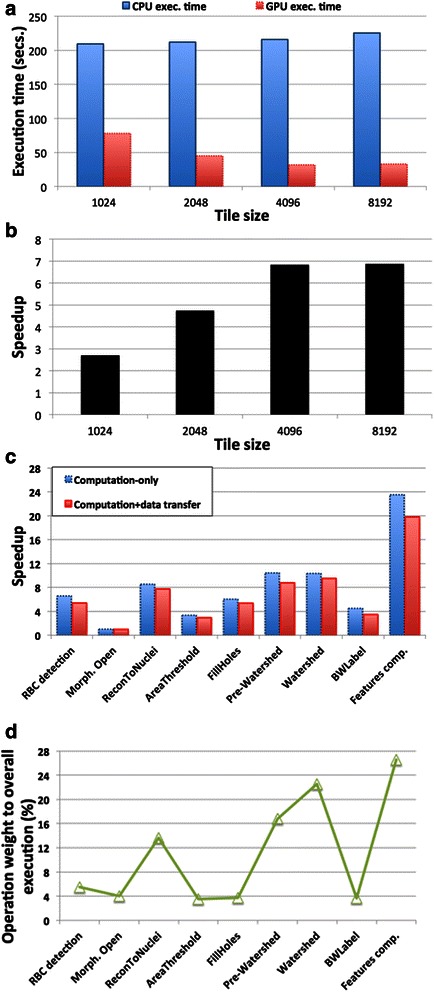
Performance improvements with the GPU-based version of the segmentation and feature computation pipeline. **a** Application execution according to tile size. **b** Application speedup according to tile size. **c** Speedup of internal operations of the application using 4Kx4K image tiles. **d** Percentage of the application execution time consumed per operation using 4Kx4K image tiles

The first set of experiments evaluates the performance of the segmentation and feature computation pipeline when the sizes of image tiles are varied. We want a tile size that results in a large number of tiles (high throughput concurrent execution across nodes and computing devices) and that leads to good speedup on a GPU. Figure 8(a) presents the execution times of the pipeline with the CPU operations and GPU-enabled operations when the image tile size is varied for an input image of 16Kx16K pixels. Figure 8(b) presents the speedup on the GPU in each configuration. We observed that tile size has little impact on the CPU execution times, but the GPU execution times decrease with larger tiles as a consequence of the larger amount of parallelism available that leads to better GPU utilization. The better GPU utilization is a result of the reduced percent in total execution time of GPU kernel launch cost/synchronization and higher data transfer rates with larger data. The analysis pipeline involves dozens of kernels, some of which are computationally inexpensive operations, such as data type transformations (from 4-byte integers to 1-byte characters), setting matrix memory to a value (memset), or device-to-device data copies. The cost of launching such kernels is high when processing small image tiles. The kernel for type transformations, for instance, takes about 77us and 864us, respectively for 1Kx1K and 4Kx4Ktiles. An operation processing a 4Kx4K image region in 1Kx1K tiles needs to call the kernel 16 times, which takes 1232us, compared to once when the same region is processed in 4Kx4K tiles. For the memset and device-to-device kernels, a single kernel call costs more or less the same for 1Kx1K and 4Kx4K tiles, making the situation worse. These performance issues are also observed in kernels with higher execution times, such as Reconstruct to Nucleus (ReconToNuclei), Fill Holes, Pre-Watershed and Watershed. The ReconToNuclei kernel, for instance, takes about 41 ms and 348 ms, respectively for 1Kx1K and 4Kx4K tiles. Processing a 4Kx4K image region in 1Kx1K tiles would result in a time cost of 656 ms. In addition to fewer kernel calls, larger image tiles lead to lower probability of thread collision. This in turn reduces the amount of serialization during atomic memory updates during the execution of a kernel. These results corroborate with other studies [102, 103] in which similar CPU/GPU relative performance trends were observed as data sizes increase. As is shown in Figs. 8(a) and (b), the 4Kx4K tile size attains the best performance (tile sizes higher than 4Kx4K did not show significant performance gains). Hence we used 4Kx4K tiles in the rest of the experiments.

Figures 8(c) and (d) present the amount of GPU acceleration for operations in the segmentation and feature computation steps and their weight to the execution of the entire pipeline, respectively. The amount of acceleration varies significantly among the operations because of their different computation patterns. The segmentation operations are mostly irregular and, as such, are likely to attain lower speedups on the GPU compared with operations with more regular data access and processing patterns. The operations that perform a flood fill execution strategy (ReconToNuclei, Fill Holes, Pre-Watershed), for instance, perform irregular data access. As described in Section 2.2, we implemented a hierarchical parallel queue data structure to improve the performance of such operations on GPUs. These operations attained similar levels of acceleration. The Pre-Watershed operation achieved slightly higher performance improvements because it is more compute intensive. AreaThreshold and BWLabel (Black and White Label), for instance, rely on the use of atomic instructions. Atomic instructions on a GPU may be costly in cases in which threads in a warp (group of threads that execute the same instruction in a lock-step) try to access the same memory address, because data accesses are serialized. This explains the low speedups attained by these operations. The operations in the feature computation phase are more regular and compute intensive. Those operations benefit from the high data throughput of a GPU for regular data access as well as the high computation capacity of the GPU.

We profiled the operations in the segmentation and feature computation steps using the NVIDIA nvprof tool to measure their efficiency with respect to the use of the GPU stream multiprocessors (sm_efficiency) and the number of instructions executed per cycle (IPC). We have chosen these metrics because most of the operations in targeted analytical pipelines execute integer operations or a mixture of integer and floating-point operations. As is presented in Table 3, the efficiency of the operation kernels is high (over 93 %), as is expected from kernels that are designed well for GPU execution. Among the kernels, regular operations, such as Red Blood Cell Detection and feature computation, achieved higher efficiency, while irregular operations, such as those based on the flood-fill scheme (see Section 2.2), tend to have a slightly lower efficiency. This is expected since the flood-fill scheme only touches pixels if they contribute to wave propagation. In this scheme, there will be few active pixels (those in the wavefront that are stored in a queue) towards the end of execution. As a result, some stream multiprocessors (SMs) have no work assigned to them. The IPC metric is higher for operations that achieve higher speedup values, as expected. For floating point operations, the maximum IPC is 2, whereas it may be higher for integer-based operations. Also, memory-bound operations tend to have smaller IPC values. We should note that the feature computation step includes several operations that mix integer and floating-point instructions. We should note that although useful in our evaluation, the reported metrics may not be useful for comparing two different algorithms -- for instance, there are different implementations of the flood fill scheme that are regular (perform raster-/anti-raster scan passes on the image). These implementations will show higher values for the reported metrics while resulting in higher (worse) execution times as compared with our implementations.

**Table 3:** Profiling information of the pipeline operations using NVIDIA nvprof tool

Pipeline Operation	Metric	
	sm_efficiency	IPC
Red Blood Cell (RBC) Detection	99.49	2.45
Morphological Open	93.12	1.41
Reconstruct to Nucleus	95.38	1.48
Area Threshold	99.30	1.05
Fill Holes	95.38	1.48
Pre-Watershed	96.35	2.13
Watershed	97.59	2.12
Black and White Label	99.86	1.12
Features Computation	98.53	3.36

We collected sm_efficiency and IPC metrics, which are the percentage of time at least one warp is active on a multiprocessor averaged over all multiprocessors on the GPU and the instructions executed per cycle, respectively

#### Cooperative execution on CPUs and GPUs

These experiments assess the performance impact when CPU cores and GPUs are cooperatively used on a computation node. Two version of the pipeline were used in the experiments: (i) 1 L refers to the version in which the operations are bundled together and each stage executes either using CPU or GPU; (ii) 2 L is the version expressed as a hierarchical pipeline with individual operations in a stage exposed for scheduling. Two scheduling strategies were compared: (i) First Come, First Served (FCFS), which does not take into account performance variability, and (ii) PATS. PATS uses the speedups presented in Fig. 8(c) for scheduling decisions.

**Fig. 9:**
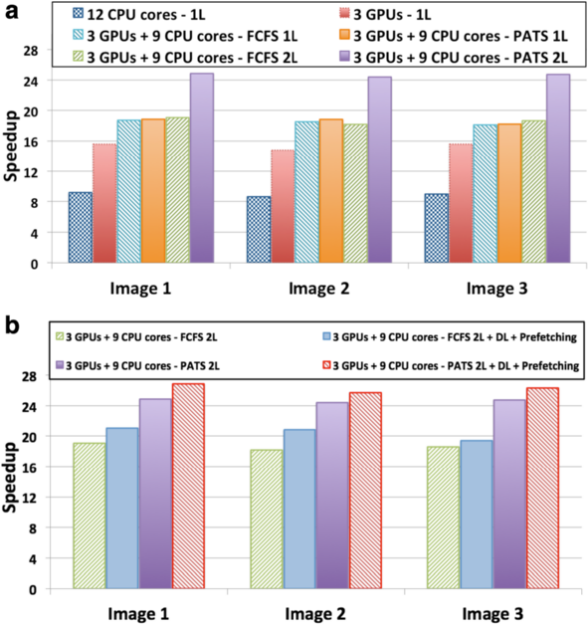
Performance of segmentation and feature Computation steps with different versions: multi-core CPU, multi-GPU, and cooperative CPU-GPU. The CPU-GPU version also evaluates the composition of the application as a single level coarse-grained pipeline (1 L) in which all stage operations are executed as a single task, and the hierarchical pipeline (2 L) in which fine-grained operations in a stage are exported to the runtime system. Additionally, FCFS and PATS scheduling strategies are used to assign tasks to CPUs and GPUs. **a** Performance of multi-core CPU, multi-GPU and cooperative CPU-GPU versions of the application. **b** Improvements with data locality (DL) mapping and asynchronous data copy optimizations

The results obtained using three randomly selected images are presented in Fig. 9(a). 3 CPU cores manage the 3 GPUs, leaving only 9 CPU cores for computation, in configurations where the CPU cores and GPUs are used together. Each GPU and each CPU core receive an image tile for processing via our scheduling strategies in these experiments; as such all of the available CPU-cores are used during execution. As is shown in the figure, the performance of the analysis pipeline improves by 80 % when the GPUs are used compared to when only CPUs are used. In the 1 L version of the application, PATS is not able to make better decisions than FCFS, because all operations in a stage are executed as a single coarse-grain task. The 2 L version using PATS improved the performance by more than 30 %. This performance gain is a result of PATS ability to maximize system utilization by assigning a task to the most appropriate device. As shown in Table 4, PATS mapped most of the tasks with high GPU speedups to GPUs and the ones with lower speedups to CPUs. The FCFS, however, scheduled 58 % of the tasks to the GPUs and 42 % to the CPUs regardless of the GPU speedup of an operation. Figure 9(b) shows the performance impact of the data locality conscious task assignment (DL) and data prefetching (Prefetching) optimizations. The 2 L version of the pipeline was used in the experiments, because the data transfer cost to execution ratio is higher compared to the 1 L version. The data transfer cost in the 1 L version is a small fraction of the execution time, because instances of the stages are scheduled as coarse-grain tasks. The optimizations improved the performance of the analysis pipeline by 10 % (when FCFS is used for task scheduling) and 7 % (when PATS is used for task scheduling). The gains are smaller with PATS because PATS may decide that it is better to download the operation results to map another operation to the GPU.

**Table 4:** Percent of tasks assigned to CPU and GPU according to the scheduling policy

Pipeline Operation	Scheduling policy			
	FCFS		PATS	
	CPU	GPU	CPU	GPU
Red Blood Cell Detection	42.7	57.3	20.2	79.8
Morphological Open	42.5	57.5	96.6	3.4
Reconstruct to Nucleus	42.6	57.4	8.9	91.1
Area Threshold	42.7	57.3	100	0
Fill Holes	43.2	56.8	89.8	10.2
Pre-Watershed	42.1	57.9	0	100
Watershed	42.2	57.8	10.2	89.8
Black and White Label	43.1	56.9	89.5	10.5
Features Computation	43.2	56.8	9.3	90.7

While FCFS assigns all pipeline operations with similar proportion to CPU and GPU, PATS preferably assigns to the GPU operations that attain higher speedups on this device. Thus, PATS better utilizes the hybrid system

#### Execution on multiple nodes

**Fig. 10:**
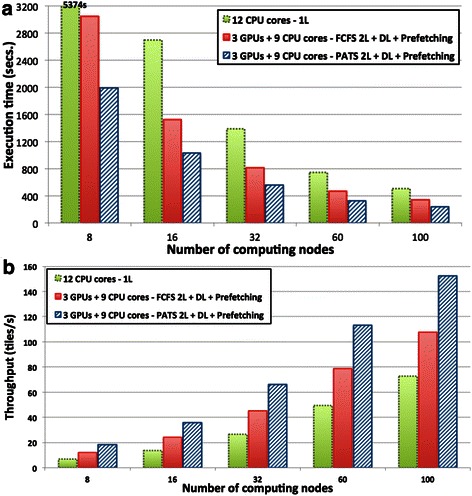
Multi-node execution of the Segmentation and Feature Computation in a strong-scaling experiment. **a** Execution times. **b** Throughput in number of tiles processed per second

These experiments evaluate the scalability of the analysis pipeline when multiple nodes of the machine are used. The evaluation was carried out using 340 Glioblastoma brain tumor WSIs, which were partitioned into a total of 36,848 4 K × 4 K tiles. The input data tiles were stored as image files on the Lustre file system. The results represent end-to-end execution of the analysis pipeline, which includes the overhead of reading input data. The execution times, as the number of nodes is varied from 8 to 100, are shown in Fig. 10(a). All implementations of the analysis pipeline achieved good speedup when more nodes are added. The performance improvements with the cooperative use of CPUs and GPUs as compared to the CPU only executions were on average 2.45x and 1.66x times with PATS and FCFS, respectively. The performance gains with the cooperative use of CPUs and GPUs are significant across the board. The analysis pipeline with CPU + GPU and PATS is at least 2.1x faster than the version that uses 12-CPU cores only. Figure 10(b) presents the throughput (tiles/s) with respect to the number of nodes. On 100 nodes with 1200 CPU cores and 300 GPUs, the entire set of 36,848 tiles was processed in less than four minutes.

### Function 3: performance of consensus clustering implementation

**Fig. 11:**
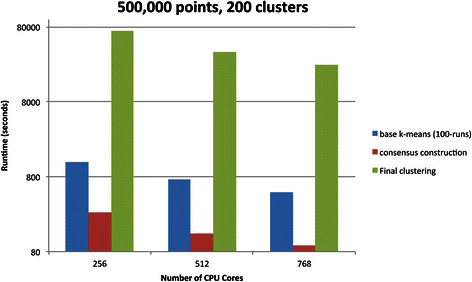
Execution times of three phases (base clustering runs, consensus matrix construction, and final clustering) in the consensus clustering process. The number of samples is 500,000. The base clustering runs and the final clustering are set to generate 200 clusters. The number of CPU cores is varied from 256 to 768. Note that the y-axis is logarithmic scale

The performance evaluation of the consensus clustering implementation was carried out on a state-of-the-art shared-memory system, called Nautilus. The Nautilus system is an SGI Altrix UV 1000 funded by the National Science Foundation to provide resources for data analysis, remote visualization, and method development. It consists of 1024 CPU cores and 4 TB global shared memory accessible through a job scheduling system. In the experiments we used a dataset with 200 million nuclei with 75 features per nucleus. We created a sample of 500,000 data points by randomly selecting nuclei from all the image tiles such that each image tile contributed the same amount of nuclei – if an image tile had fewer nuclei than necessary, all of the nuclei in that image tile were added to the sampled dataset. The execution times of the base clustering, consensus matrix construction, and final clustering phases of the consensus clustering process are shown in Fig. 11. The number of clusters was set to 200 in the experiments. As is seen from the figure, the execution times of all the phases decrease as more CPU cores are added – speedup values of 2.52, 2.76, and 2.82 are achieved on 768 cores compared to 256 cores. The memory consumption on 768 cores was about 1.1 TB including space required for the data structures used by the k-means algorithm.

## Conclusions

Grading cancer specimens is a challenging task and can be ambiguous for some cases exhibiting characteristics within the various stages of progression ranging from low grade to high. Innovations in tissue imaging technologies have made it possible for researchers and clinicians to collect high-resolution whole slide images more efficiently. These datasets contain rich information that can complement information from gene expression, clinical, and radiology image datasets to better understand the underlying biology of disease onset and progression and to improve the diagnosis and grading process. However, the size of the datasets and compute-intensive pipelines necessary for analysis create barriers to the use of tissue image data. In our work we have identified three core functions to support more effective use of large datasets of tissue images in research and clinical settings. These functions are implemented through a suite of efficient methods as well as runtime frameworks that target modern high performance computing platforms.

The capacity to search and compare the morphology and staining characteristics across imaged specimens or within a given tissue sample is extremely valuable for assisting investigators and physicians who are charged with staging and classifying tissue samples. The methods of Function 1 (CBIR) enable this capacity. They can be used to generate image-based feature signature of unclassified imaged specimens with the profiles of a set of “gold-standard” cases and enable automatic retrieval of those samples exhibiting the most similar morphological and staining characteristics – in the case of prostate cancers, to deliver the computed Gleason score and confidence interval to the individual seeking support. Likewise investigators can provide a representative sample within a given imaged specimen and use the methods to quickly detect and locate other sub-regions throughout the specimen, which exhibit similar signatures. Our team is currently building an *ImageMiner* portal for diverse histopathology image analysis and applications, which includes medical image segmentation, CBIR, and registration. Upon completion the portal will be made available as open source to the research and clinical communities.

The methods and tools of Functions 2 and 3 are critical to building the capacity for analyses with very large tissue image datasets. Our work has demonstrated that high data processing rates can be achieved on modern HPC systems with CPU-GPU hybrid nodes. This is made possible by employing techniques that take into account variation in GPU performance of individual operations and implement data reuse and data prefetching optimizations. Shared memory systems provide a viable platform with large memory space and computing capacity for the classification stage when it is applied on segmented objects.

## Availability of data and materials

The experiments for the high performance computing software tools used datasets publicly available from The Cancer Genome Atlas repository (https://tcga-data.nci.nih.gov/tcga/). The source codes for the analysis pipelines in these experiments are released as a public open source through the following links: https://github.com/SBU-BMI/nscale and https://github.com/SBU-BMI/region-templates.

### Ethics

The work presented in this manuscript is focused on the development of software tools and methods. We have used publicly available datasets and de-identified datasets approved by the Institutional Review Boards for the respective grants: 5R01LM011119-05, 5R01LM009239-07, and 1U24CA180924-01A1.

### Consent

This work is not a prospective study involving human participants.

### Availability of supporting data

The datasets used in the high performance computing experiments are publicly available from The Cancer Genome Atlas repository (https://tcga-data.nci.nih.gov/tcga/).

## Footnotes

1
http://nvidia.com/cuda

2
https://github.com/SBU-BMI/nscale

3
https://github.com/SBU-BMI/region-templates
